# Does Educational Mismatch Affect Emigration Behaviour?

**DOI:** 10.1007/s10680-021-09595-z

**Published:** 2021-10-20

**Authors:** Philippe Wanner, Marco Pecoraro, Massimiliano Tani

**Affiliations:** 1 Institute of Demography and Socioeconomics, University of Geneva, Geneva, Switzerland; 2 Institute of Economic Research, University of Neuchatel, Neuchatel, Switzerland; 3 University of New South Wales, Canberra, Australia

**Keywords:** Emigration, Return migration, Onward migration, Wages, Occupation, Educational mismatch

## Abstract

This paper uses linked Swiss administrative and survey data to examine the relationship between educational mismatch in the labour market and emigration decisions, carrying out the analysis for both Swiss native and previous immigrant workers. In turn, migrants’ decisions separate returning home from onward migration to a third country. We find that undereducation is positively associated with the probability of emigration and return to the country of origin. In contrast, the reverse relationship is found between overeducation and emigration, especially among non-European immigrant workers. According to the predictions of the traditional model of migration, based on self-selection, migrants returning home are positively selected relative to migrants emigrating to other countries. We also find that immigrants from a country outside the EU27/EFTA have little incentive to return home and generally accept jobs for which they are mismatched in Switzerland. These results highlight the relevance to understand emigration behaviours in relation to the type of migrant that is most integrated, and productive, in the Swiss market, hence enabling better migration and domestic labour market policy design.

## Introduction

In today’s labour market, there are increasing differences between the actual skills of the workforce and the requirements of the economy, as the tasks associated with each occupation change rapidly due to technology, automation, and globalization among others. Consequently, the incidence of educational and skills mismatches is not uncommon. A mismatch between a worker’s skills and their job may arise in the form of educational mismatch (also referred to as vertical mismatch),^1^ or/and horizontal mismatch, which refers to a situation where the skills acquired in a specific field or occupation differ from those required by the job carried out.

Educational mismatches are associated with reduced levels of satisfaction at work (e.g. Allen & van der Velden, 2001) and wages (e.g. Leuven & Oosterbeek, 2011; McGuinness, 2006). Overeducation and skills mismatches have also been associated with higher job mobility (e.g. Grunau & Pecoraro, 2017; McGoldrick & Robst, 1996; Pecoraro, 2014) and higher interregional or international migration, particularly among workers that are overeducated in their home labour market (Quinn & Rubb, 2005; Villarreal, 2016).

For those who have already migrated, a poor match in the host countries’ labour market gives an incentive to re-migrate. As suggested by Borjas and Bratsberg (1996), the decision to migrate depends on the expected wage premium from investing in a (temporary or permanent) stay in the “new” labour market. Alternatively, if upward career mobility does not occur or is not fast enough, workers may be tempted to search for, and take up when possible, relatively more prestigious or high-paying jobs available in other regions or countries (Sicherman & Galor, 1990). While these arguments can be identified in theoretical models of migration choices, their empirical analysis is constrained by a general lack of detailed information about emigrants and their characteristics. In fact, the relationship between educational mismatch and immigration has attracted a large literature which is predominantly carried out from the viewpoint of the destination country, with no study about the links between migrants’ educational mismatches and their subsequent emigration choices (Piracha & Vadean, 2013).

This paper aims to address this gap by investigating the relationship between vertical mismatches and the international mobility of both migrants and natives living in Switzerland. The Swiss labour market is particularly interesting as migrants represent one third of its workforce, include both skilled and unskilled individuals, and earn wages that are among the highest in the world (Wanner et al., 2016). Uniquely, our analysis benefits from novel individual-level administrative and survey data, which link four registers and surveys and enable us to distinguish the streams of migrants returning home from that of migrants moving onward to another country. These data are not only scarce but also comprehensive, as the declaration of emigration, which includes the country of destination, cancels the obligation to pay hefty Swiss health insurance premia, and, in some cases, give access to the old-age pension.

Throughout the paper, we apply the terms ‘emigration’ and ‘outmigration’ interchangeably to natives and immigrants living in Switzerland deciding to migrate and leave the country. Our objective is to estimate to what extent mismatches experienced in Switzerland can be seen as a catalyst to seek better fortunes in another country. As a result, we only consider individuals in working age and not close to retirement. Educational-occupational mismatches are based on observed education and job performed. Similarly to most studies of educational mismatch, the excess/deficit in education for a migrant is measured as if his/her education is acquired in Switzerland, as information on where education is acquired is not available.

## Review of the Literature

### Educational Mismatches

Educational mismatch defines a state where a person is employed in a job that requires a lower amount of education than what that person has actually acquired (Leuven & Oosterbeek, 2011). Educational mismatches are more commonly characterizing migrants rather than natives (Aleksynska & Tritah, 2013; Lindley, 2009; Prokic-Breuer & McManus, 2016; Piracha & Vaeden, 2013), though there are wide variations depending on the country of origin considered (Visintin et al., 2015).

The factors explaining migrants’ higher rates of mismatches include the imperfect international transferability of human capital (Chiswick & Miller, 2009; Tani et al., 2013), itself due to the fact that some of the human capital acquired through education and work experience is country-specific. Other explanations are discrimination in the destination labour market (Battu & Sloane, 2004; Nielsen, 2011), inadequate language skills (Green et al., 2007; Prokic-Breuer & McManus, 2016), and a poor educational-occupational match prior to migration (Nowotny, 2016; Piracha et al., 2012). By international standards, Switzerland has low rates of educational mismatch (Quintini, 2011), though its incidence of overeducation reflects the high proportion of migrants in its population (Pecoraro, 2011).

### Theoretical Framework: Roy’s Model of Self-Selection

The literature typically views migration as the result of an individual decision that involves comparing expected costs and benefits of staying versus migrating. This approach builds on Roy’s model (see Borjas, 1987), which suggests that if earnings reflect individual productivity, then international differences in average wages and income inequality will exert a different pull on differently able and motivated individuals. As a result, the choice to migrate is influenced by both salaries available in the home country and those expected in the country of destination. As salaries vary across a variety of observed characteristics such as gender and educational level as well as unobserved features like motivation, both observed and unobserved determinants influence natives’ and migrants’ sorting across and within labour markets. Hence, if migration is not restricted and salaries are relatively higher abroad, emigration will occur. Similarly, when home conditions are superior to those available elsewhere, people will tend to stay in their places of residence.

### Determinants of Emigration

On the basis of Roy’s model, an educational mismatch, by itself, could cause emigration when an individual concludes that opportunities in another country are better than what offered in the place of residence (Jensen & Pedersen, 2007; Reagan & Olsen, 2000). One would therefore expect to find mismatched natives wanting to emigrate as well as poorly matched immigrants. While the case for emigration for the natives is clear, the situation is more complex in the case of existing migrants, as they have a choice of either returning home, hence completing their migration experience, or move to a third country in search of better fortune.

The literature purports that returning home is a viable option for two opposite types of migrants (Borjas & Bratsberg, 1996): those who have achieved their migration goals, for instance, in terms of accumulated experience and earnings potential in a foreign labour market, and those who failed to integrate in the labour market for example because they are unemployed (Pecoraro, 2012), earn lower wages compared to their reference group (Huber & Nowotny, 2016; Stark & Taylor, 1991), or face tougher than expected conditions in the host country (Caron & Ichou, 2020; Constant & Massey, 2002).

Among the types of migrants considering returning home, gender plays a distinct role. In general, immigrant women in industrialized countries are less likely to return home relative to men, as they are better off in terms of status and social position vis-a-vis their country of origin, particularly if this is a low-income country (Bachmeier et al., 2013). Women are also characterized by more frequent family migration than men (Coulter et al., 2011; Ette et al., 2016; Pecoraro, 2012). In Switzerland, 40.5% of women migrants indicate family reasons are one of the reasons to migrate compared to 19.5% for men (Wanner, 2019).

With reference to possibly move to a third country, the literature highlights the integration level of migrants and indirectly their settlement intentions (Mak, 1997; Steinmann, 2019), along with the social dimension of their integration (Ette et al., 2016), measured for instance by language skills and their social network in the host country (van Dalen and Henkens 2008), and the number of years of residence in the host country (Dustmann, 1996; van Baalen & Müller, 2009). Typically, the higher the duration of stay the lower is the probability of further outmigration. Having a settlement permit, which is granted after a few years in Switzerland, is generally regarded as a deterrent in further emigration (Pecoraro, 2012; Wanner, 2020).

For both natives and existing immigrants, emigration is more likely the higher the level of human capital. Highly educated natives and migrants are expected to be more mobile than their less educated equivalent, as they can use their skills in an international labour market and have a better access to information (Coulter et al., 2011; Sapeha, 2017). However, the association between the human capital and emigration is not always linear (Pungas et al., 2012). Risk-aversion also plays a role in limiting the propensity to emigrate for natives, as they will invariably face more uncertainty in a host country relative to their place of origin (Pecoraro, 2012; Zlotnik, 2006).

### Return or Onward Migration

The limited literature studying the determinants of emigration does not distinguish between onward and return migration. As Monti (2020) mentions, “studies of re-emigration have often failed to appropriately distinguish emigration types”. However, with the increasing capacity to be mobile, a deeper attention was made not only on return migration but also on onward migration (Jeffery & Morrison, 2011). Onward migration is increasing in a transnational world. Many working-age immigrants are onward emigrants, and this proportion is higher for migrants from Eastern Europe, Africa and Asia than for those from Northern and Western Europe and North America; onward emigration is also higher among highly educated migrants than among their low-educated peers (Nekby, 2006; Schroll, 2009), and refugees (Monti, 2020).

The factors explaining return migration are generally regarded as distinct from those explaining onward migration. A positive association is observed between return migration and distance between host and home countries (Bratsberg and al., 2007) as well as the human capital acquired by migrants (Dustmann & Weiss, 2007). Unemployment is also found to lead more frequently to onward than to return migration (Nekby, 2006) though there seems to be a type of U-shape in the proportion of returns among emigrants according to age, with a higher proportion being younger than 30 and older than 55 years (Larramona, 2013). A deterioration of the socio-economic conditions in the host country can also lead to a secondary move (Mas Giralt, 2017).

The relationship between the choice to emigrate and educational mismatch has rarely been studied (Quinn & Rubb, 2005, 2011; Villarreal, 2016). There is no evidence regarding the impact of “poor integration” in the labour market, as proxied by educational mismatch, on return versus onward migration. Our paper aims at shedding light on this topic.

## Aims and Hypotheses

Unlike many other destination countries for immigrants, Switzerland collects multiple sources of microdata that can be jointly combined to study migration decisions (Steiner & Wanner, 2015) and is characterized by high levels of both immigration and emigration in an area of free movement of persons (Fioretta & Wanner, 2016; Steiner, 2019), as well as political and economic stability.

During the period under study, Switzerland was characterized with a low level of unemployment (less than 5%) and positive economic growth, offering employment opportunities in the labour market. In this context, failure or success in the labour market is better expressed by educational mismatches rather than by labour market participation.

Wages are higher in Switzerland than in most of the industrialized countries, a factor that may deter natives from emigration, though emigrating Swiss people generally move to countries with lower wages, possibly masking the effect of expatriates relocating within multinational corporations.

According to the literature, the impact of educational mismatch should vary not only depending on the type of emigration but also according to the country of origin. In particular, different results are also expected depending on whether or not the migrant belongs to the regime of free circulation of persons prevalent in the EU27/EFTA region, which includes Switzerland. Therefore, based on the literature, we test the following three hypotheses:

### Hypothesis 1

**A failure in terms of labour market integration**, which here takes form of overeducation, leads to an increase in emigration compared to the case of little or no educational mismatch (Borjas & Bratsberg, 1996) for both natives and EU27/EFTA migrants. Natives will be encouraged to leave Switzerland in the event of challenging conditions in the labour market. Migrants that are mismatched in Switzerland are unlikely to find suitable jobs in surrounding countries with similar economic, social, and political conditions. Thus, their return migration is expected to be more frequent than onward migration after controlling for different factors such as age, sex, or country of origin.

### Hypothesis 2

**A failure in terms of labour market integration for non-EU27 migrants** living in Switzerland is less likely to be accompanied by a return home, where they will have to accept lower average wages in their places of origin. We also expect that migrants who do not hold a permanent residence permit will prefer to stay in Switzerland despite poor labour market integration in order to secure or maintain their residence status. Furthermore, access to another EU27/EFTA country will be limited given that non-EU citizens have restricted access to the European labour market because they are not part of the policy of free movement of persons. Hence, they face more difficulty with international mobility relative to EU27/EFTA migrants, and as a result, be less inclined to re-emigrate (Bodvarsson et al., 2014).

### Hypothesis 3

**A success in terms of labour market integration** leads to onward migration for both natives and foreign-born workers under the hypothesis that these workers are the most adaptable and are more likely to find a job in the global–international–labour market (Kuhlenkasper & Steinhardt, 2018). They will probably choose a country where firms offer them higher earnings if they decide to emigrate. Alternatively, due to their skill endowments, they may join the brain circulation of professional elites (Salt, 1992; Johnson & Reget, 1998). Therefore, they are likely to exhibit an increased probability of onward migration and a decreased probability of return migration.

Additional hypotheses are the following: The effects of mismatch on emigration should be heterogeneous not only with respect to the country of origin but also according to other relevant characteristics such as gender, age, and education. Women, particularly those who come from countries in transition or in development, are expected to be less influenced by their position in the labour market compared to men: they often find in Switzerland better conditions in terms of gender perceptions and expectations than in other countries. Moreover, they more frequently migrate for family reasons and less frequently for labour purposes (Wanner, 2019), and their return is therefore conditioned by an appreciation of both family and professional impacts.

According to the human capital theory, the expected benefits of migration are higher for younger persons due to a longer duration of stay in the destination country (Bodvarsson et al., 2015). It is also expected that younger migrants are more likely to emigrate in cases of failure or success than older migrants, with career mobility being higher in early career stages (McCue, 1996).

Finally, emigrants should be positively selected in terms of education (Nekby, 2006; Wasmer et al., 2006). Put differently, the highly educated (compared to those with a low or intermediate levels of education) are expected to exhibit higher mobility in the international labour market in the case of either failure or success in the national labour market.

## Data

For the empirical analysis, we use an innovative dataset based on the combination of four sources of information (Steiner & Wanner, 2015): (1) the Swiss Population Register, which is exhaustive and provides information on different demographic dimensions and the date of arrival or departure of every person living in Switzerland, (2) the Social Security Register, which provides the yearly professional income of every individual working in the Swiss labour market, (3) the Structural Surveys for years 2010–2015 and (4) the Swiss unemployment register, which informs on unemployment periods for every individual active in the labour market. The Structural Surveys cover more than 200,000 individuals per year aged 15 and over. This survey takes place on December 31 of every year and gathers information on dimensions related to demography, education, occupation and language skills. The questionnaire is available in ten languages. The four sources were linked using the Social Security Number, which is available on statistical sources since 2010 and allows deterministic matching. Once matched, these sources enable us to identify, for each surveyed person, possible emigration during the years following the survey. Emigration is recorded in the Swiss Population Register.

The focus is on a sub-sample of persons born abroad and living in Switzerland at the time of the surveys. As we are interested in potential earnings over the period under study, we limit the analysis to persons who declared they were full-time salaried workers at the time of the survey (assuming that this information is also valid for the rest of the year). Among the persons interviewed by the Structural Survey aged 20–59, 93% of men and 57% of women report working full-time at the time of the survey, but we do not know the percentage of (average) time spent working at other points of time. To avoid persons leaving Switzerland for retirement reasons, we only retain workers aged 20–59. To perform comparative analyses, we also run separate models for natives, in order to evaluate whether the factors behind emigration are the same according to the migratory status.

The data are not covering border workers living in neighbouring countries, neither illegal workers; however, for a country such as Switzerland, their anticipated number is low (Morlok and al., 2015).

Information on professional yearly earnings is available for every person working legally in Switzerland through the Social Security Register. This source of information provides the total individual income from employment, as well as the period covered by social security contributions. When using the income variable, one needs to take into account that the work status (part-time or full-time) during the whole year is not known.

Episodes of unemployment are estimated through the Unemployment Register, which provides information on the type of unemployment (full-time or partial) for the years 2010–2016.

The final sample consists of 175,902 individuals born abroad and 348,029 natives, aged 20–59, with valid information on earnings and working full-time at the time of the survey.

Immigration towards Switzerland is based on different permits, the most frequent being the resident permit (C-permit) and the annual permit (B-permit), respectively. The C-permit allows the migrant to stay in Switzerland almost indefinitely, while the B-permit is renewable every year depending on the availability of a job. In general, migrants holding an annual permit are more frequently characterized by circular or “serial” migration (Ossman, 2004; Zufferey, 2019). The residence permit is conditional to a quasi-permanent stay, not allowing departure for a period of more than 6 months. It therefore permits but also encourages permanent residence in Switzerland.

In order to control for the impact of the permit on remigration, the foreign born were split into two groups based on those permits: Holders of an annual permit (B-permit, *n* = 54,663) and holders of a resident permit (C-permit), including naturalized migrants (*n* = 121,239).^2^

### Emigration Status

The emigration status is the dependent variable, which is provided by the Swiss Population Register. When migrating out of the country, persons must declare their destination to the administration. However, this obligation is not always effective, but financial incentives to do so are high, as the declaration of emigration cancels the obligation to pay for Swiss health insurance and in some case to benefit from old-age pension abroad. Therefore, the country of destination is known for approximately 90% of emigrants, and onward or return migrations can be distinguished. As suggested by the OECD (2008), we used the country of birth rather than the last country visited or the country of citizenship as an indicator of the country of origin to measure the frequency of return migration. Thus, return migration is defined as migration of the foreign born towards the country of birth. Onward migration is defined as migration to a country other than the country of birth. Return migration is of course not considered for natives.

We focused on the departure observed during the 12 months following the Structural Survey. In our sample, among the Swiss born, 1,250 (0.3%) left the country during this period. The number of permanent permit holders leaving Switzerland was 964 (0.8%), and the number of annual permit holders leaving was 1,469 (5%). To validate those proportions, we computed the emigration rates among the whole population aged 20–59 living in Switzerland: the rates in 2017 were of 0.5% (Swiss born), 1.5% (holders of a permanent permit) and 8% (holders of an annual permit), respectively. The sample of the Structural Survey therefore underestimates the actual level of emigration among foreign-born individuals. People who are planning to leave Switzerland in the weeks or months following the survey feel less obligation to complete the survey. It is assumed that the participants surveyed before their departure are, however, representative of all those who leave, which leads us to believe that there is no specific bias associated with this under-representation.

Among the foreign born who emigrated, a majority returned to their country of origin (see Table 1). This is particularly the case among holders of an annual permit.

**Table 1:** Distribution of the sample according to emigration status, place of origin and permit

		Emigration		Return		Onward	
	Total sample	*N*	%	*N*	%	*N*	%
Swiss Born	348,029	1250	0.34				
Foreign-Born, resident permit	121,239	964	0.79	507	0.43	398	0.32
Foreign-Born, annual permit	54,663	2,169	3.91	1,469	2.72	605	1.04

Own computations based on structural survey and STATPOP statistics. Proportions are weighted

### Educational Mismatch

The Structural Survey requests information on the highest level of education achieved and the current occupation carried out. Educational mismatch is identified by comparing the years of schooling (estimated based on the achieved education) with the modal years of education required for the occupation (Visintin and al., 2015; Verdugo & Verdugo, 1989). Years of required education correspond to the workers’ modal years of actual education in each ISCO occupation (3-digit level) and by age group (20–29/30–39/40–49/50–59). The same measurement method was also implemented using the Swiss standard classification of professions (SBN code, 3-digit^3^) rather than the ISCO code.

There is no mismatch if the number of years of education corresponds to the modal value calculated for salaried workers in the same occupation. Overeducation is then identified among workers having a higher number of years of schooling than the modal years of schooling within each occupation.^4^ In contrast, undereducation refers to a situation in which the person is employed in a job that generally requires more years of education than achieved. This traditional measure of mismatch is then used to predict the emigration behaviour of both migrants and natives.

Table 2 provides the distribution of educational mismatch for the selected sample, according to the migration status and the two typologies of occupations (ISCO or SBN). Both typologies provide almost the same results, even if some discrepancies are observed at the individual level: in total, 13.3% of the native born and 10.2% of the foreign born are classified differently depending on the typology considered. Based on the ISCO typology, 54% of the natives are adequately educated (56% when using the SBN typology), and this proportion is 36% among the foreign-born population (37% with SBN typology). Among the natives, 23% are overeducated, a proportion that reaches 37% among the foreign born. The remaining 21% (natives) and 26% (foreign born) are undereducated.

**Table 2:** Distribution of the occupational mismatch status for the sample and the native population aged 20–59 according to the typology used

	Using the SBN 3-digit code to estimate required education			
	Over educated	Adequately educated	Under educated	Total
*Panel A: Swiss born*				
Using the ISCO 3-digit code				
Overeducated	66,242	9886	5976	82,104
	***19.0***	*2.8*	*1.7*	*23.6*
Adequately educated	10,401	172,974	10,318	193,693
	*3.0*	***49.7***	*3.0*	*55.7*
Undereducated	4289	5621	62,241	72,151
	*1.2*	*1.6*	***17.9***	*20.7*
Total	80,932	188,481	78,535	347,948
	*23.3*	*54.2*	*22.6*	*100.0*
*Panel B: Foreign born*				
Using the ISCO 3-digit code				
Overeducated	60,289	2227	1817	64,333
	***34.3***	*1.3*	*1.0*	*36.6*
Adequately educated	3034	57,082	5035	65,151
	*1.7*	***32.5***	*2.9*	*37.1*
Undereducated	1680	4168	40,498	46,346
	*1.0*	*2.4*	***23.0***	*26.4*
Total	65,003	63,477	47,350	175,830
	*37.0*	*36.1*	*26.9*	*100.0*

Weighted proportions. Own computations based on structural survey and STATPOP statistics. Proportions are indicated in italics. Information that is consistent between both concepts are indicated in bold

Models were run for both typologies to test the sensitivity of the model according to the measure of educational mismatch. The results obtained were very close and led to the same interpretation. Therefore, only the results based on the ISCO code are presented here.

Table 3 presents the incidence of educational mismatch for migrant and native workers after taking into account the place of origin and the type of permit. Data are weighted according to the weights provided by the Swiss Federal Statistical Office, which is in charge of the Structural Survey. The situation of the migrant population on the labour market is less favourable compared to that of natives in terms of overeducation: 20% of natives are overeducated. Almost 24% of the foreign born holding a permanent permit are in the same situation, and at least 30% among those holding an annual permit. These figures are more or less similar by gender, the discrepancy in terms of overeducation incidence being the highest among migrants with an annual permit (35% for women versus 29% for men). As the recent immigration flows towards Switzerland are characterized by a demand for highly educated labour in specific sectors of activity, those migrants are generally better integrated in the labour market compared with those who arrived during the 1990s (Wanner et al., 2016). The proportion of undereducated workers is 23% among the Swiss, 40% among foreigners holding a permanent permit or who are naturalized and 29% among those holding an annual permit. Systematically, non-EU27/EFTA residents are characterized less by undereducation and more by overeducation than EU27/EFTA residents. Gender differences are not prevalent among the Swiss born and UE27/EFTA migrants, but significant among non-UE27/EFTA ones, where undereducation is lower and overeducation higher among women compared to men.

**Table 3:** Proportion of undereducated and overeducated workers according to the place of birth, the gender, the permit, the region of origin and the type of measurement

		Swiss born	Foreign born					
			C-permit (permanent)^1^			B-Permit (annual)		
			Total	EU27/EFTA	Non-EU27/EFTA	Total	EU27/EFTA	Non-EU27/EFTA
*ISCO*								
Undereducated	Total	23.2	40.0	37.7	42.9	29.2	27.0	35.1
	Men	22.8	39.7	37.4	42.7	31.5	29.1	37.7
	Women	24.3	40.7	38.3	43.3	24.4	22.6	29.4
Overeducated	Total	20.5	23.7	25.3	21.8	30.9	31.2	29.9
	Men	21.1	23.3	24.9	21.3	28.8	29.2	27.9
	Women	19.0	24.5	26.0	22.7	35.2	35.5	34.2
*SBN*								
Undereducated	Total	22.9	40.2	37.5	43.5	30.2	28.0	36.4
	Men	21.7	39.0	36.6	42.3	31.7	29.3	38.2
	Women	25.9	42.6	39.6	46.0	27.1	25.3	32.3
Overeducated	Total	22.3	24.3	26.0	22.1	31.2	31.8	29.8
	Men	23.4	24.4	26.1	22.1	29.4	29.9	28.2
	Women	19.7	24.1	25.7	22.1	35.0	35.6	33.3
*Sample size*								
ISCO		348,029	121,239	69,606	51,633	54,663	40,279	14,384
SBN		347,948	121,188	69,589	51,599	54,642	40,263	14,379

Weighted proportions. Sample size can vary according to some missing values. Own computations based on structural survey and STATPOP statistics. ^1^ including naturalized migrants

Based on the factors of emigration identified in the literature, control variables included in the analyses are the following:

- Sex (men–reference category, women),
- Age and age squared,
- Level of education (lower secondary–reference category, upper secondary, tertiary),
- Country of birth: EU27/EFTA countries—reference category, other European countries, non-European countries that are members of the OECD, non-European countries not members of the OECD,
- Civil status (single, married–reference category, not married),
- Region of residence in Switzerland (7 regions, Zurich being the reference category),
- Type of municipality (centre of agglomeration–reference category, other agglomeration municipality, isolated city, rural municipality)
- Language skills, using two variables. The first one indicates the main language (German or Swiss German dialect, French, Italian, English, other). The second one determines the number of languages spoken by the migrant (one, two or more languages),
- Year of the survey (2010 to 2015)
- Status of employment: (employed during the last five years–reference, unemployed during the last five year, partially unemployed during the last five year)
- For the foreign born only, the number of years since migration (and its square).
- Unfortunately, we were not able to control for all relevant confounding factors due to the lack of information on some of them, such as the reason for immigration to Switzerland or the family composition, as well as the ties between Switzerland and the country of birth.
- Taking into account those confounding factors allows us to estimate how educational mismatch influences the probability of leaving Switzerland. Two variables were introduced in the model: the years of overeducation and the years of undereducation.

## Model

In a first step, logistic regressions were run to measure the association of different factors identified in the literature with outmigration, without distinguishing between return and onward migration. Logistic regressions are used to explain the probability (*p*) of educational mismatch according to the dimensions under study and different control variables (Cox et Snell 1989). The formula is as follows:

$${\text{logit}}\left( {\text{p}} \right) = {\text{ln}}({\text{p}}/\left( {{1} - {\text{p}}} \right) = \beta \_0 + \beta \_{\text{1 x}}\_\left( {{\text{i}},{1}} \right) + \beta \_{\text{2 x}}\_\left( {{\text{i}},{2}} \right) + \cdots$$

where *i* denotes an individual unit, *β*_0 is a constant and *β*_(1,…n) are the coefficients of the explanatory variables *x*_(1,… n).

For all models, the levels of significance (* *p* < 0.10; ** *p* < 0.05; ***p < 0.01) are presented to facilitate the interpretation of the results.

In a second step, we use a multinomial logit regression model to identify the determinants of outmigration. This model is appropriate to express the probability of return or onward migration according to our two indicators of educational mismatch and other individual characteristics, the alternative being to remain in Switzerland. We assume that outmigration for individual *i* is determined by the following model: *M*_i_* = *O*_*i*_ α + *X*_*i*_ β + *e*_*i*_, with *i* = 1, 2,…, *N*. *O* is a vector including the two variables referring to educational mismatch, *X* is a vector including control variables and *e* is a normally distributed term reflecting the unobservable component of outmigration. For the foreign born, given that the latent model is not observed, we define the variable *M* as the realization of three possible states:

$$M_{i} = j = \left\{ {\begin{array}{*{20}c} 1 \\ 2 \\ 3 \\ \end{array} \begin{array}{*{20}c} \\ \\ \\ \end{array} \begin{array}{*{20}l} {{\text{if }}M_{i } < \mu_{1 } } \hfill \\ {{\text{if }}\mu_{1} \le M_{i } < \mu_{2 } } \hfill \\ {{\text{otherwise}}{.}} \hfill \\ \end{array} \begin{array}{*{20}l} {({\text{no outmigration)}}} \hfill \\ {({\text{return migration)}}} \hfill \\ {({\text{onward migration)}}} \hfill \\ \end{array} } \right.$$

The probabilities that individual *i* will be in situation *j* are:

$${\mathbb{P}}\left( {M_{i} = j{|}O_{i} ,X_{i} } \right) = \frac{{{\text{exp}}\left( {O_{i} \alpha_{j} + X_{i} \beta_{j} } \right)}}{{\mathop \sum \nolimits_{h = 1,2,3 } \exp \left( {O_{i} \alpha_{h} + X_{i} \beta_{h} } \right)}}$$

To explore the potential heterogeneity in the effects of educational mismatch, separate models are estimated for migrants from EU27/EFTA countries or from other countries, and for annual permit holders versus holders of other permits (long-term permit or naturalized foreign born). For comparative purposes, the model was also computed for Swiss natives. In this case, the duration of stay, language indicators and the place of birth were not introduced in the model. Because return migration is not prevalent for Swiss natives, only onward migration was considered as emigration.

To measure the consistency of our measurement of educational mismatch and because salary data are available, we also run models using the individual income as a dependent variable (log-wage). Although the relationship between possible overeducation and a salary penalty is not the main subject of our study, the analysis is conducted in order to check the consistency of our measure of educational mismatch, since overeducation has been shown to be associated with a wage penalty in the literature.

Finally, it should be noted that the relationship being estimated is not necessarily causal but serves to identify important correlations in the data.

## Results

We discuss first the results from the logistic model, which does not distinguish between the two types of emigration (return vs. onward), including all countries of birth and natives. In a second step, we focus on migrants and distinguish between return and onward migration.

### Educational Mismatch and Emigration: All Countries of Birth and Natives Separately

**Fig. 1:**
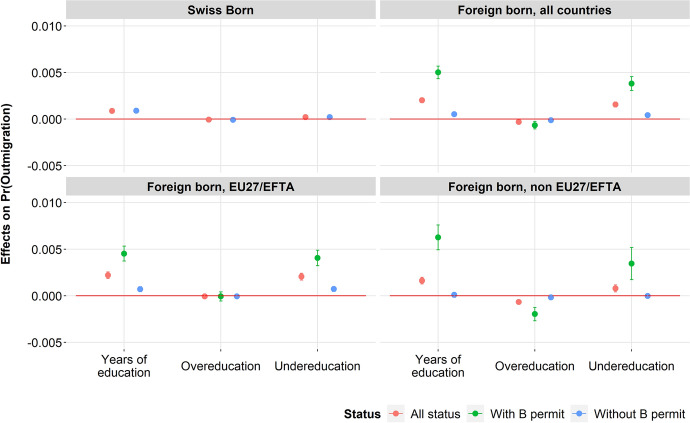
Marginal effects of the number of years of education, overeducation and undereducation on the probability of outmigration among Swiss and foreign-born

Table 4 shows the beta coefficients derived from the logit models explaining outmigration for the selected sample of full-time salaried workers and separately according to the place of birth (natives and foreign-born). Figure 1 shows the average marginal effects of the years of actual education, years of overeducation and years of undereducation on the probability of outmigration according to the place of origin and the status. Before focusing on the impacts of educational mismatch on outmigration, the results are briefly commented on for the other variables included in the models.

**Table 4:** Logit results for the determinants of outmigration, only full-time salary workers, from 2010 to 2015 (marginal effects and standard error)

	Swiss born		All countries of birth						Born in EU27/EFTA countries					
			All statutes		Other permits		Annual permit		All statutes		Other permits		Annual permit	
Years of actual education	0.262***	(0.025)	0.104***	(0.015)	0.037	(0.027)	0.131***	(0.019)	0.092***	(0.017)	0.042	(0.031)	0.115***	(0.021)
**Educational mismatch**														
Well-matched (reference)														
Years of overeducation	− 0.021	(0.018)	− 0.020*	(0.010)	− 0.024	(0.021)	− 0.017	(0.011)	− 0.007	(0.011)	− 0.022	(0.024)	− 0.002	(0.013)
Years of undereducation	0.062***	(0.020)	0.091***	(0.016)	0.055*	(0.027)	0.099***	(0.020)	0.097***	(0.018)	0.074*	(0.031)	0.103***	(0.022)
**Level of education**														
Compulsory education (reference)														
Upper secondary education	− 0.820***	(0.190)	0.234*	(0.103)	0.208	(0.170)	0.264*	(0.132)	0.247*	(0.124)	0.311	(0.217)	0.243	(0.154)
Tertiary education	− 1.362***	(0.266)	0.2	(0.159)	0.525*	(0.267)	0.077	(0.200)	0.202	(0.188)	0.555*	(0.327)	0.076	(0.233)
**Status of residence**														
Swiss nationals (reference)														
C-permit (permanent)	− 0.548***	(0.190)	− 0.087	(0.162)	− 0.17	(0.168)			− 0.17	(0.212)	− 0.09	(0.218)		
B-permit (annual)	1.050***	(0.345)	0.366*	(0.159)					0.144	(0.207)				
Naturalized	0.084	(0.115)	0.074	(0.188)	0.069	(0.191)			− 0.158	(0.260)	− 0.064	(0.266)		
**Years since migration**			− 0.119***	(0.009)	− 0.121***	(0.012)	− 0.119***	(0.020)	− 0.125***	(0.011)	− 0.126***	(0.014)	− 0.129***	(0.028)
**Years since migration squared**			0.002***	0.000	0.002***	0.000	0.002	(0.002)	0.002***	0.000	0.002***	0.000	0.001	(0.003)
**Country of birth**														
UE/EFTA (reference)														
Other Europe			− 0.566***	(0.099)	− 0.727***	(0.163)	− 0.531***	(0.127)						
OECD rest of the world			0.512***	(0.088)	0.068	(0.203)	0.636***	(0.100)						
Non-OECD rest of the world			0.083	(0.067)	− 0.297*	(0.133)	0.195*	(0.079)						
**Sex**														
Men (reference)														
Women	0.023	(0.066)	− 0.111*	(0.045)	0.096	(0.085)	− 0.186***	(0.052)	− 0.105*	(0.052)	0.093	(0.099)	− 0.175***	(0.060)
**Age**	− 0.058*	(0.025)	− 0.111***	(0.019)	− 0.197***	(0.033)	− 0.078***	(0.024)	− 0.125***	(0.023)	− 0.229***	(0.040)	− 0.085***	(0.028)
**Age squared**	0	0.000	0.001***	0.000	0.002***	0.000	0.001***	0.000	0.001***	0.000	0.003***	0.000	0.001***	0.000
**Marital status**														
Married (reference)														
Single	0.005	(0.082)	0.109*	(0.050)	0.092	(0.104)	0.108*	(0.057)	0.003	(0.057)	− 0.015	(0.118)	− 0.004	(0.066)
Other marital status	0.523***	(0.121)	− 0.063	(0.091)	− 0.043	(0.142)	− 0.073	(0.120)	− 0.118	(0.107)	0.004	(0.164)	− 0.198	(0.143)
**Type of municipality**														
Centre of agglomeration (reference)														
Other agglomeration municipality	− 0.120*	(0.070)	− 0.109*	(0.045)	− 0.146*	(0.087)	− 0.090*	(0.053)	− 0.114*	(0.053)	− 0.218*	(0.100)	− 0.073	(0.062)
Isolated city	− 1.741*	(0.711)	− 0.073	(0.293)	− 0.363	(0.580)	0.051	(0.339)	− 0.132	(0.329)	− 0.478	(0.703)	− 0.016	(0.373)
Rural municipality	− 0.519***	(0.095)	− 0.202***	(0.068)	− 0.240*	(0.121)	− 0.174*	(0.082)	− 0.198***	(0.075)	− 0.321*	(0.139)	− 0.141	(0.090)
**Region of residence**														
Zurich (reference)														
Geneva Lake	0.492***	(0.090)	0.115*	(0.066)	− 0.031	(0.141)	0.150*	(0.075)	0.071	(0.081)	− 0.214	(0.165)	0.152	(0.093)
Mitteland	− 0.161	(0.104)	− 0.149*	(0.080)	− 0.087	(0.149)	− 0.185*	(0.097)	− 0.235*	(0.094)	− 0.307*	(0.174)	− 0.220*	(0.112)
Nord-West	− 0.227*	(0.119)	− 0.017	(0.073)	0.105	(0.139)	− 0.062	(0.086)	− 0.018	(0.084)	0.072	(0.159)	− 0.054	(0.100)
East	− 0.326*	(0.130)	0.109	(0.082)	0.285*	(0.150)	0.043	(0.099)	0.119	(0.091)	0.246	(0.172)	0.079	(0.108)
Centre	− 0.197	(0.124)	0.103	(0.080)	0.086	(0.161)	0.112	(0.093)	0.126	(0.091)	0.052	(0.180)	0.153	(0.105)
Ticino	− 0.286*	(0.161)	− 0.094	(0.126)	− 0.237	(0.243)	− 0.045	(0.146)	− 0.118	(0.143)	− 0.484*	(0.285)	0.003	(0.164)
**Surveyed in:**														
2010 (reference)														
2011	− 0.02	(0.096)	0.118*	(0.068)	0.127	(0.147)	0.117	(0.077)	0.086	(0.080)	0.184	(0.172)	0.065	(0.091)
2012	− 0.326***	(0.105)	− 0.053	(0.072)	0.036	(0.147)	− 0.071	(0.083)	− 0.037	(0.084)	0.074	(0.172)	− 0.054	(0.096)
2013	− 0.127	(0.099)	0.012	(0.069)	0.178	(0.139)	− 0.036	(0.081)	0.027	(0.081)	0.166	(0.165)	− 0.004	(0.094)
2014	− 0.176*	(0.102)	0.009	(0.070)	0.053	(0.141)	0.01	(0.081)	0.06	(0.081)	0.117	(0.165)	0.065	(0.094)
2015	− 0.136	(0.106)	0.016	(0.070)	0.105	(0.142)	0.005	(0.082)	0.05	(0.081)	0.094	(0.166)	0.059	(0.094)
**Status of employment**														
Employed during the last 5 years (reference)														
Full Unemployment during the last 5 years	0.121	(0.088)	− 0.215***	(0.068)	0.049	(0.106)	− 0.353***	(0.093)	− 0.152*	(0.081)	− 0.064	(0.133)	− 0.187*	(0.104)
Partial unemployment during the last 5 years	− 0.141	(0.293)	− 0.812*	(0.443)	− 1.02	(0.748)	− 0.662	(0.547)	− 0.849	(0.555)	− 0.486	(0.750)	− 1.330*	(0.750)
**Main language**														
Other language (reference)														
German			0.094	(0.066)	− 0.08	(0.147)	0.165*	(0.075)	0.228***	(0.083)	− 0.177	(0.189)	0.348***	(0.093)
French			− 0.155*	(0.075)	− 0.101	(0.161)	− 0.157*	(0.086)	− 0.002	(0.091)	− 0.075	(0.194)	0.018	(0.105)
Italian or Romansh			− 0.102	(0.108)	− 0.081	(0.207)	− 0.113	(0.125)	− 0.039	(0.120)	− 0.13	(0.237)	− 0.039	(0.140)
English			0.522***	(0.078)	0.575***	(0.184)	0.496***	(0.086)	0.651***	(0.105)	0.744***	(0.230)	0.611***	(0.118)
**Multilingualism**														
Speaking only one language (reference)														
Two languages			− 0.148*	(0.058)	− 0.209*	(0.112)	− 0.125*	(0.068)	− 0.161*	(0.071)	− 0.230*	(0.137)	− 0.129	(0.084)
Three or more languages			− 0.523***	(0.108)	− 0.581*	(0.229)	− 0.507***	(0.123)	− 0.730***	(0.152)	− 0.650*	(0.308)	− 0.753***	(0.176)
Number of observations	348,029		142,992		88,418		54,574		96,464		56,245		40,219	

	Swiss born		Born in non-EU27/EFTA countries					
			All statutes		Other permits		Annual permit	
Years of actual education	0.262***	(0.025)	0.133***	(0.033)	− 0.004	(0.061)	0.179***	(0.040)
**Educational mismatch**								
Well-matched (reference)								
Years of overeducation	− 0.021	(0.018)	− 0.054***	(0.018)	− 0.029	(0.041)	− 0.055***	(0.021)
Years of undereducation	0.062***	(0.020)	0.070*	(0.036)	− 0.015	(0.059)	0.097*	(0.051)
**Level of education**								
Compulsory education (reference)								
Upper secondary education	− 0.820***	(0.190)	0.02	(0.198)	0.018	(0.274)	0.084	(0.287)
Tertiary education	− 1.362***	(0.266)	0.098	(0.308)	0.627	(0.500)	− 0.091	(0.415)
**Status of residence**								
Swiss nationals (reference)								
C-permit (permanent)	− 0.548***	(0.190)	− 0.236	(0.263)	− 0.182	(0.270)		
B-permit (annual)	1.050***	(0.345)	0.631*	(0.250)				
Naturalized	0.084	(0.115)	0.263	(0.276)	0.291	(0.283)		
**Years since migration**			− 0.114***	(0.015)	− 0.130***	(0.023)	− 0.097***	(0.031)
**Years since migration squared**			0.002***	0.000	0.003***	0.000	0.001	(0.002)
**Country of birth**								
UE/EFTA (reference)								
Other Europe								
OECD rest of the world			0.926***	(0.139)	1.016***	(0.292)	0.966***	(0.165)
Non-OECD rest of the world			0.523***	(0.113)	0.459*	(0.208)	0.598***	(0.144)
**Sex**								
Men (reference)								
Women	0.023	(0.066)	− 0.139	(0.089)	0.117	(0.165)	− 0.233*	(0.105)
**Age**	− 0.058*	(0.025)	− 0.080*	(0.038)	− 0.123*	(0.059)	− 0.052	(0.051)
**Age squared**	0	0.000	0.001*	0.000	0.002*	(0.001)	0.001	(0.001)
**Marital status**								
Married (reference)								
Single	0.005	(0.082)	0.349***	(0.098)	0.388*	(0.226)	0.306***	(0.112)
Other marital status	0.523***	(0.121)	0.085	(0.172)	− 0.151	(0.288)	0.206	(0.219)
**Type of municipality**								
Centre of agglomeration (reference)								
Other agglomeration municipality	− 0.120*	(0.070)	− 0.089	(0.088)	0.085	(0.178)	− 0.139	(0.102)
Isolated city	− 1.741*	(0.711)	0.185	(0.635)	− 0.141	(1.016)	0.424	(0.820)
Rural municipality	− 0.519***	(0.095)	− 0.294*	(0.159)	0.045	(0.244)	− 0.521*	(0.217)
**Region of residence**								
Zurich (reference)								
Geneva Lake	0.492***	(0.090)	0.168	(0.118)	0.435	(0.279)	0.092	(0.132)
Mitteland	− 0.161	(0.104)	0.089	(0.159)	0.478*	(0.286)	− 0.083	(0.200)
Nord-West	− 0.227*	(0.119)	− 0.027	(0.146)	0.184	(0.292)	− 0.117	(0.170)
East	− 0.326*	(0.130)	− 0.005	(0.196)	0.426	(0.308)	− 0.212	(0.279)
Centre	− 0.197	(0.124)	0.017	(0.172)	0.196	(0.356)	− 0.053	(0.200)
Ticino	− 0.286*	(0.161)	− 0.075	(0.266)	0.348	(0.293)	− 0.246	(0.329)
**Surveyed in:**								
2010 (reference)								
2011	− 0.02	(0.096)	0.215	(0.131)	− 0.016	(0.284)	0.280*	(0.150)
2012	− 0.326***	(0.105)	− 0.101	(0.144)	− 0.1	(0.282)	− 0.095	(0.168)
2013	− 0.127	(0.099)	− 0.021	(0.137)	0.202	(0.262)	− 0.098	(0.164)
2014	− 0.176*	(0.102)	− 0.153	(0.138)	− 0.186	(0.273)	− 0.137	(0.162)
2015	− 0.136	(0.106)	− 0.073	(0.140)	0.107	(0.273)	− 0.107	(0.165)
**Status of employment**								
Employed during the last 5 years (reference)								
Full Unemployment during the last 5 years	0.121	(0.088)	− 0.282*	(0.122)	0.314*	(0.179)	− 0.712***	(0.189)
Partial unemployment during the last 5 years	− 0.141	(0.293)	− 0.725	(0.728)			0.103	(0.751)
**Main language**								
Other language (reference)								
German			− 0.281*	(0.144)	0.128	(0.261)	− 0.474*	(0.197)
French			− 0.459***	(0.144)	− 0.211	(0.300)	− 0.512***	(0.172)
Italian or Romansh			0.211	(0.290)	0.44	(0.295)	0.162	(0.365)
English			0.332***	(0.114)	0.181	(0.292)	0.303*	(0.123)
**Multilingualism**								
Speaking only one language (reference)								
Two languages			− 0.054	(0.105)	− 0.216	(0.214)	− 0.053	(0.122)
Three or more languages			− 0.21	(0.166)	− 0.565	(0.362)	− 0.117	(0.189)
Number of observations	348,029		46,528		31,736		14,355	

Structural Survey (2010, 2011, 2012, 2013, 2014, 2015), Swiss Population Register and Social Security Register. Notes: Coefficient estimates, robust standard errors in parentheses; ***p < 0.01, **p < 0.05, *p < 0.10; data are weighted. Those without valid information on the outcome and control variables are excluded

In line with the literature, sex slightly affects the probability of outmigration among foreign-born individuals. It does not significantly change the probability of outmigration among natives, but being a woman appears to significantly decrease the probability of outmigration for both EU27/EFTA and non-EU27/EFTA natives holding an annual permit. It does not influence the probability for holders of a permanent permit. As only full-time salaried workers are included, the sample generally contains women without young children: recent mothers generally work part-time in Switzerland (Lacroix & Vidal Coso, 2018). Our results suggest that international mobility for full-time salaried native women is almost at the same level than for men, after having taken into account all confounding factors. Such a result is not surprising, as international migration is now balanced between men and women (UN, 2017).

Age is also significantly associated with the outmigration of foreign-born individuals, as well as civil status. Indeed, being single increases the probability of outmigration among the foreign born, while being divorced or widowed leads to an increase in outmigration among the native born. The type of municipality of residence also plays a role in the sense that people living in rural municipalities migrate less. In contrast, inhabitants of the Lake Geneva area are more mobile, which can be easily explained by the presence in this region of international organizations and multinational enterprises.

Having experienced a situation of unemployment during the five years preceding the survey (and then having found a full-time job by the time of the survey) slightly decreases the probability of outmigration. This means that re-integration in the labour market after a period of unemployment intensifies attachment to the host country.

English-speaking migrants are more likely to emigrate than those speaking another foreign language. In contrast, the probability of outmigration is reduced for French speakers. This result can be explained by the relative lack of professional opportunity in France compared to Switzerland as well as the discrepancy in terms of working conditions between Switzerland and France. Foreign-born individuals with bilingual proficiency show a decreased probability of outmigration. This can most likely be explained by the fact that for those foreign-born individuals, one of the spoken languages is often a language officially spoken in Switzerland^5^; this indicates a good level of linguistic integration, which generally occurs when the foreign born wish to stay in the host country. It is therefore not surprising that those foreign-born individuals are less likely to leave the country.

After taking these confounding factors into account, the level of education plays a significant role in the probability of leaving the country, which differs according to the place of birth. Among natives, less educated persons are more mobile internationally. Among foreign-born individuals, holding an upper secondary or tertiary education (compared to a lower secondary level) increases the probability of outmigration for migrants. This result confirms those obtained in previous studies (e.g. Barbiano di Belgiojoso & Ortensi, 2013; Nekby, 2006; Pecoraro, 2012).

Among foreign-born workers, the type of permit plays a significant role in outmigration, as holders of an annual permit are more mobile. This is, of course, explained by the fact that this permit is conditioned on the availability of a job in Switzerland. It is also the permit that is attributed during the first years of one’s stay in Switzerland, a period characterized by rather high mobility. The place of birth also influences the probability to emigrate: native-born individuals from non-EU27/EFTA European countries (most of them being from former Yugoslavian countries and Turkey) are less mobile than the reference category, as those born in OECD countries outside Europe are more mobile.

After taking into account those factors, the number of years of actual education is significantly associated with mobility; that is, the more years of education, the higher the probability of outmigration. The average marginal effects are the highest among annual permit holders, who are also the more mobile group (Fig. 1). The years of actual education, on the contrary, do not significantly influence the probability of outmigration among permanent permit holders of non-EU27/EFTA countries.

Being undereducated significantly increases the probability of outmigration among both the native born and foreign born (except those from non-EU27/EFTA countries with permanent permits). This result can be easily explained by the fact that the undereducated workers perform well not only in the Swiss labour market but also abroad. For this reason, they are mobile in the labour market and likely to be employed in other countries, compared to the reference category of workers who are not undereducated. Overeducated workers are no more mobile than those who are adequately educated. In contrast, international mobility is hampered for those from non-EU27/EFTA countries with annual permit.

### Educational Mismatch and Onward/Return Outmigration: All Countries of Birth

**Fig. 2:**
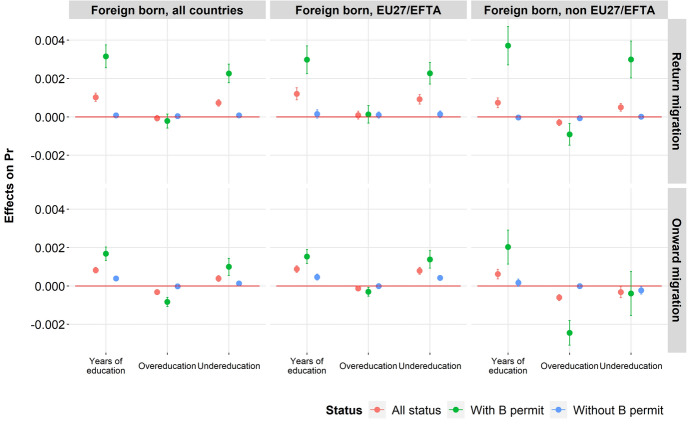
Marginal effects of the number of years of education, overeducation and undereducation on the probability of return and onward migration among foreign-born

Table 5 and Fig. 2 show the results for the same models after subdividing outmigration between return and onward migration. Only the foreign-born population is considered, as the distinction between onward and return migration is not relevant for the native-born population. The impacts of the confounding factors included in the models are generally consistent with those presented above and are not described here.

**Table 5:** Multi-Logit results for the determinants of return and onward migration, only full-time salaried workers born outside Switzerland, from 2010 to 2015 (marginal effects and standard error)

	All countries of birth				Born in EU27/EFTA countries				Born in non-EU27/EFTA countries			
	Return		Onward		Return		Onward		Return		Onward	
Years of actual education	0.094***	(0.019)	0.137***	(0.027)	0.083***	(0.021)	0.136***	(0.032)	0.147***	(0.043)	0.117*	(0.050)
**Educational mismatch**												
Well-matched (reference)												
Years of overeducation (1st def)	− 0.019	(0.012)	− 0.024	(0.016)	− 0.011	(0.014)	0.004	(0.020)	− 0.046*	(0.025)	− 0.069*	(0.027)
Years of undereducation (1st def)	0.093***	(0.018)	0.100***	(0.037)	0.092***	(0.020)	0.141***	(0.044)	0.115***	(0.042)	0.01	(0.075)
**Level of education**												
Compulsory education (reference)												
Upper secondary education	0.146	(0.122)	0.608***	(0.220)	0.187	(0.142)	0.757***	(0.284)	− 0.355	(0.269)	0.406	(0.363)
Tertiary education	0.094	(0.193)	0.682*	(0.308)	0.114	(0.221)	0.830*	(0.401)	− 0.128	(0.408)	0.58	(0.493)
**Country of birth**												
UE/EFTA (reference)												
Other Europe	− 0.906***	(0.134)	− 0.032	(0.165)								
OECD rest of the world	0.570***	(0.106)	0.462***	(0.159)					1.257***	(0.183)	0.472*	(0.228)
Non-OECD rest of the world	− 0.339***	(0.094)	0.621***	(0.112)					0.390*	(0.160)	0.616***	(0.168)
Number of observations	142,855		142,855		96,366		96,366		46,489		46,489	

Structural Survey (2010, 2011, 2012, 2013, 2014, 2015), Swiss Population Register & Social Security Register. Notes: Coefficient estimates, robust standard errors in parentheses; ***p < 0.01, **p < 0.05, *p < 0.10; data are weighted. Those without valid information on the outcome and control variables are excluded. Results obtained after control of the following variables: status of residence, years since migration, sex, age, marital status, type of municipality, region of residence, year of the survey, status of employment, main language, multilingualism

Considering all foreign-born individuals and introducing the place of birth as a variable in the model significantly influences the likelihood of onward/return migration. Compared to migrants from the EU27/EFTA, those from non-European/OECD countries are more likely to migrate back home or to another country. Such workers are generally highly qualified and stay in Switzerland temporarily, often in the context of multinational relocations, which can explain their mobility.

On the other hand, the probability of return and onward migration is lower among migrants from other European countries (not members of EU27/AELE). For these migrants, the probability of return is also lower, as the probability of onward migration is significantly increased. Those migrants have fewer incentives to return to their country of origin when they are of working age because their earnings potential is lower back home, while migration costs are substantial due to immigration restrictions. The results are therefore in line with the literature (see, for instance, Borjas & Bratsberg, 1996).

After taking into account the confounding factors, the models indicate that the higher the level of actual education, the greater the probability of both return and onward mobility. However, for migrants with a permanent permit, there is not a significant difference in the probability of leaving Switzerland associated with the number of years of actual education. This result is certainly explained by the fact that this group stays in Switzerland for a longer time and is less mobile, regardless of actual education.

Compared to foreign-born workers whose actual education corresponds to the requirements of their occupation, those who are overeducated do not show an increased or decreased probability of emigrating, either to another country or to the country of origin. The only significant result refers to a slightly lower probability of onward migration among holders of an annual permit from non-EU27/EFTA countries. Considering the permit these individuals hold, it can be assumed that they have recently arrived in Switzerland and that in accordance with the theory of career mobility (Sicherman & Galor, 1990), they accept a professional sacrifice, probably linked to the non-immediate transferability of their skills, while anticipating a future improvement in their professional situation.

The results are different for undereducated migrants. When they hold an annual permit, they are characterized by increased mobility, either to another country or to return home. For resident permit holders, however, this increase in the probability of leaving is not observed.

### Robustness Checks

To check the robustness of our analyses, we first computed the same models based on the Swiss typology of occupations, which differs from the ISCO code provided by the ILO for the purpose of international comparisons. As indicated in Table 2, the classification of workers according to under- or overeducation status differs for approximately 13% of natives and 10% of those foreign-born. These robustness checks in this context aim at verifying whether the models provide the same results, regardless of the typology adopted.

**Fig. 3:**
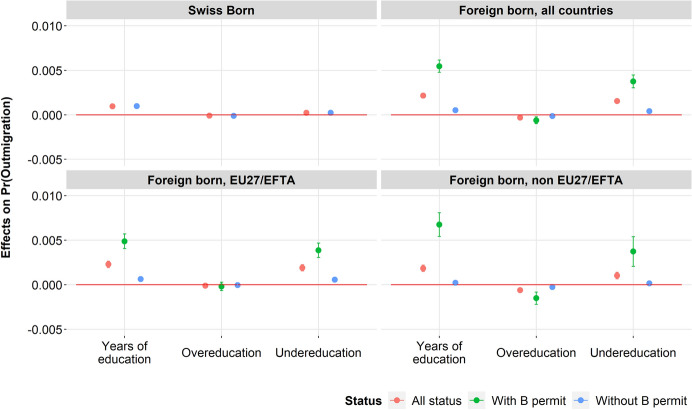
Marginal effects of the number of years of education, overeducation and undereducation on the probability of outmigration among Swiss and foreign-born, according to the SBN typology of occupations

**Fig. 4:**
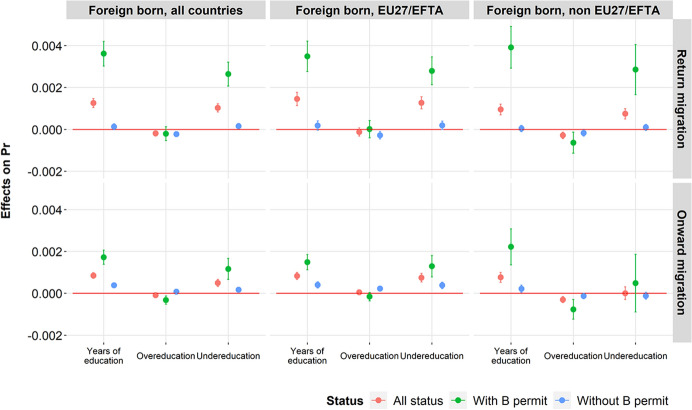
Marginal effects of the number of years of education, overeducation and undereducation on the probability of return and onward migration among foreign-born, according to the SBN typology of occupations

Figure 3 shows the average marginal effects of outmigration estimated after taking into account the different confounding variables. Figure 4 shows the average marginal effects after distinguishing the type of outmigration. As expected, the results are almost the same–the magnitude of the estimates and the confidence intervals are close regardless of the typology–and lead to similar interpretations.^6^

Second, consistent with the overeducation literature, we examine the relationship between mismatch and wages to evaluate the consistency of our measure in relation to various forms of emigration. Table 6 shows the impact of educational mismatch on the wages of full-time salaried workers. Overall, the results obtained are coherent as expected. After taking into account the confounding factors, overeducation is linked to a significant decrease in wages. In contrast, undereducation is associated with a positive return in terms of wages. These results are observed for both natives and foreign born and for the different groups of foreign born defined according to the region of origin and the permit. The results are always significant (*p* < 0.01).

**Table 6:** OLS results for the determinants of log-wage, only full-time salary workers, from 2010 to 2015 (marginal effects and standard error)

	Swiss born		All countries of birth						Born in EU27/EFTA countries					
			All statutes		Other permits		Annual permit		All statutes		Other permits		Annual permit	
Years of actual education	0.076***	(0.001)	0.072***	(0.001)	0.073***	(0.001)	0.069***	(0.002)	0.077***	(0.001)	0.080***	(0.002)	0.072***	(0.002)
**Educational mismatch**														
Well-matched (reference)														
Years of overeducation (1st def)	− 0.019***	(0.001)	− 0.037***	(0.001)	− 0.036***	(0.001)	− 0.037***	(0.001)	− 0.028***	(0.001)	− 0.026***	(0.002)	− 0.030***	(0.002)
Years of undereducation (1st def)	0.037***	0.000	0.051***	(0.001)	0.051***	(0.001)	0.051***	(0.002)	0.053***	(0.001)	0.055***	(0.001)	0.050***	(0.002)
**Level of education**														
Compulsory education (reference)														
Upper secondary education	0.123***	(0.005)	0.024***	(0.004)	0.021***	(0.005)	0.015*	(0.008)	0.023***	(0.006)	0.038***	(0.007)	0.001	(0.010)
Tertiary education	0.127***	(0.008)	0.195***	(0.009)	0.185***	(0.010)	0.201***	(0.016)	0.150***	(0.011)	0.163***	(0.013)	0.136***	(0.018)
**Country of birth**														
UE/EFTA (reference)														
Other Europe			− 0.084***	(0.004)	− 0.090***	(0.004)	− 0.063***	(0.009)						
OECD rest of the world			− 0.013	(0.012)	− 0.038*	(0.016)	0.005	(0.019)						
Non-OECD rest of the world			− 0.164***	(0.005)	− 0.181***	(0.006)	− 0.148***	(0.008)						
Number of observations	347,099		140,360		87,311		53,049		94,712		55,510		39,202	

	Swiss born		Born in non-EU27/EFTA countries					
			All statutes		Other permits		Annual permit	
Years of actual education	0.076***	(0.001)	0.058***	(0.002)	0.056***	(0.002)	0.058***	(0.004)
**Educational mismatch**								
Well-matched (reference)								
Years of overeducation (1st def)	− 0.019***	(0.001)	− 0.059***	(0.002)	− 0.056***	(0.003)	− 0.060***	(0.003)
Years of undereducation (1st def)	0.037***	0.000	0.042***	(0.002)	0.040***	(0.002)	0.047***	(0.004)
**Level of education**								
Compulsory education (reference)								
Upper secondary education	0.123***	(0.005)	0.035***	(0.006)	0.032***	(0.007)	0.021	(0.015)
Tertiary education	0.127***	(0.008)	0.309***	(0.016)	0.266***	(0.017)	0.372***	(0.034)
**Country of birth**								
UE/EFTA (reference)								
Other Europe								
OECD rest of the world			0.105***	(0.014)	0.119***	(0.017)	0.077***	(0.022)
Non-OECD rest of the world			− 0.069***	(0.006)	− 0.062***	(0.006)	− 0.081***	(0.011)
Number of observations	347,099		45,648		31,801		13,847	

Structural Survey (2010, 2011, 2012, 2013, 2014, 2015), Swiss Population Register & Social Security Register. Notes: Coefficient estimates, robust standard errors in parentheses; ***p < 0.01, **p < 0.05, *p < 0.10; data are weighted. Those without valid information on the outcome and control variables are excluded. Years of required education correspond to the workers’ modal years of actual education in each ISCO occupation (3-digit level) and by age group (20–29/30–39/40–49/50–59) among full-/part-time salary workers who are Swiss nationals or foreigners with C- and B-permits. Weighted proportions. Source: Own computations based on Structural survey, Social Register and STATPOP Statistics. ^1^ including naturalized migrants. Results obtained after control of the following variables: status of residence, years since migration, sex, age, marital status, type of municipality, region of residence, year of the survey, status of employment, main language, multilingualism

Among overeducated persons, the wage penalty is the highest for the foreign born from non-UE/EFTA countries and the lowest among the natives. The impact of an additional year of overeducation is three times higher for non-UE27/EFTA migrants than for natives. Workers from EU27/EFTA countries are in between. Years of undereducation lead to the most significant increase in wages among EU27/EFTA migrants and the lowest increase among natives.

## Discussion

Educational mismatch is a common issue in industrialized countries. Based on a meta-analysis of more than 180 studies, Leuven and Oosterbeek (2011) indicate that 30% of the workforce is overeducated and 26% is undereducated. The extent of overeducation declined from the 1970s to the 1990s but increased during the first decade of the twenty-first century. Based on original data, this paper studies how educational mismatch among migrants and natives is associated with the probability of outmigration, by distinguishing–for the foreign born–between returning to the country of origin and onward migration. This study relies on the availability of relevant information on the country of emigration in the Swiss Population Register and the linkage between this register and the Swiss Structural Survey, the latter providing detailed information on actual education and occupation. Moreover, other linkages with the Social Security Register and the Unemployment Register increase the uniqueness of the matched data by providing information on individual earnings and episodes of unemployment. As a whole, the linked data provide a rich variety of information about migrants and natives that allowed us not only to precisely define and validate the concept of educational mismatch but also to estimate the propensity to emigrate after controlling for a large range of confounding factors for a large sample of the population.

Emigration flows are generally difficult to document based on registers, as emigrants do not always declare their departure. However, for the categories of migrants covered by this study, incentives to declare are important: at the arrival in Switzerland, every inhabitant has to contract an insurance with a Swiss health insurance company. This insurance is mandatory for natives or migrants with a medium- and long-term permit. The only exceptions refer to undocumented migrants, international civil servants and embassy staff, who are not included in this study. Health insurance being expensive (more than 4000 euros per year), the incentive to declare the departure is high. Another incentive is linked to the possibility of keeping one's old-age pension (sometimes to receive them directly at the time of departure). We are however aware that a few numbers of emigrants will keep a residence in Switzerland and remain registered as resident (e.g. in order to keep their health insurance to benefit from Swiss health system in case of illness). This can lead to a slight underestimation of emigration flows.

Another specificity of our analysis refers to the measure of educational mismatch, based on the comparison between the years of schooling and the modal years of education required for the occupation by age cohorts. This measurement method assumes that a migrant will compare his/her situation with the one of the general population and not with the one of his/her own community. This assumption makes sense for the groups under study, even if migrants, in particular those arriving from non-EU27/EFTA countries, are aware of the fact the general population is not always a valid benchmark.

Many studies analyse emigration without distinguishing return and onward migration. This choice is generally explained by the lack of data regarding the country where the emigrant moves. The few studies considering this distinction conclude that the determinants of both types of emigration differ considerably, particularly for non-EU citizens. Therefore, accounting for onward and return migration separately is of crucial importance to understand how the lack of integration in the labour market will affect migrants’ outmigration behaviour. Based on the existing studies and theories on return migration, our hypothesis state that a failure of labour market integration, expressed in terms of overeducation, leads to an increase in emigration for workers who belong to the free movement of persons area (natives and EU27/EFTA migrants) because these workers can easily leave Switzerland for another labour market where they can find positions that better correspond to their skills. However, this hypothesis is not verified, and two reasons can explain why: On the one hand, the workers may have unmeasured incentives to stay in Switzerland, such as higher wages than in foreign countries or more attractive working conditions. On the other hand, they may also anticipate an improvement in their professional status in the medium term. Finally, other elements such as family factors, can also act as barriers to mobility.

Undereducated migrants are, by contrast, more mobile, particularly those holding an annual permit. Those coming from EU27/EFTA countries tend to move to third countries, meaning that they are more sensitive to their labour market outcomes in the host country. Such results may be explained by the free movement for EU27/EFTA workers that significantly increases international mobility and allows immigration to ‘grease the wheels’ of the Swiss labour market. Non-EU migrants have fewer work opportunities in Europe and are not characterized by onward migration. Therefore, even if they are in a situation of overeducation, they tend to stay in Switzerland, where they find working conditions that are better than those expected in the country of origin. Probably migrants in Switzerland not only consider their own situation in the host country, but as Engzell and Ichou (2020) suggested, also judge their success in the host country after having taken into account their social position before migration and compare themselves to their counterparts in the country of origin (see for instance Zuccotti et al., 2017).

Undereducation, by contrast, increases their probability of returning in line with the Roy model of self-selection. As a whole, it should be noted that the causality of the relationship between emigration and mismatch is not clear: educational mismatch can, in certain cases, stem from a voluntary choice to work in Switzerland with the objective of returning home or emigrating to a third country after some months or years. It can also be the consequence of non-work-related migration–for example, dictated by the desire to accompany a family or join a partner–and thus be accepted by the worker, who finds other advantages to staying in Switzerland.

Finally, mobility depends on the residence permit. An annual permit favours mobility, while a settlement permit significantly reduces it. This result is explained by the fact that the settlement permit characterizes people who have been living in Switzerland for a long period, and mobility decreases with the length of residence.

### The Region of Origin Impacts Mobility

Only EU27/EFTA citizens have full access to the Swiss labour market according to the free movement of persons. They also have better opportunities, compared to other foreigners, concerning the recognition of diplomas and professional mobility across cantons. Migrants arriving from a country outside the EU27/EFTA do not have access to the free movement of persons, and their stay in Switzerland depends on their professional activity. Those from countries in transition (for instance, the Balkan countries) have little incentive to return home due to the lack of occupational opportunities in their home country. Put differently, they generally accept occupations for which they are mismatched in Switzerland, as the anticipated situation in their country of origin is probably worse in terms of earnings, job security or working conditions. Moreover, for some groups, the political situation in the country of origin can make a return difficult.

The Swiss labour market is relatively open and is thus influenced by the surrounding countries. This opening mainly attracts low-educated and highly qualified workers, or those who have been successful in their occupations. Therefore, in terms of economic policy implications, our results demonstrate the need to rethink how to retain the most successful workers in the Swiss labour market.

## Footnotes

1 Vertical mismatch is usually measured in terms of over/undereducation or over/underskilling.

2 The permanent residence permit is accessible after 5 years of residence for EU27/EFTA citizens and 10 years for non-EU27/EFTA citizens.

3 https://www.bfs.admin.ch/bfs/en/home/statistics/work-income/nomenclatures/sbn2000.html.

4 Educational mismatch was also computed by considering the modal number of years of education by origin group (native, migrant), to test the hypothesis that migrants compare not only to the overall full-time salaried workforce, but also to the population of their origin. Because the results based on this alternative computation are qualitatively similar, they are not presented here and are available upon request.

5 Switzerland has four official languages: German, French, Italian and Romansh.

6 Complete results are not presented on this paper but are available on request.

## Declarations

### Code availability

Available.

### Data availability

The data used are the property of the Swiss Federal Statistical Office and according to the contract of data protection is not available for other researchers.
